# MicroRNA regulation in pulmonary fibrosis: a bibliometric analysis of global research trends, collaborative networks, and emerging frontiers

**DOI:** 10.3389/fmed.2026.1914903

**Published:** 2026-08-10

**Authors:** Fan Wu, Qile He, Fengjun Zhang, Xing Zhang, Wei Zhang

**Affiliations:** 1 Department of Pulmonary Diseases, Shuguang Hospital Affiliated to Shanghai University of Traditional Chinese Medicine, Shanghai, China; 2 Department of Pulmonary Diseases, Shuguang Hospital, Shanghai Institute of Infectious Diseases and Biosecurity, Shanghai University of Traditional Chinese Medicine, Shanghai, China; 3 Zhang Wei Baoshan Famous Traditional Chinese Medicine Inheritance Studio, Shanghai Baoshan Hospital of Integrated Chinese and Western Medicine, Shanghai, China; 4 Institute of Infectious Diseases, Shanghai Institute of Traditional Chinese Medicine, Shanghai, China

**Keywords:** bibliometric analysis, extracellular vesicles, MicroRNAs, pulmonary fibrosis, research trends

## Abstract

**Background:**

Pulmonary fibrosis, particularly idiopathic pulmonary fibrosis (IPF), is a progressive and fatal interstitial lung disease with limited treatment options. MicroRNAs (miRNAs) have emerged as critical regulators of fibrotic processes, yet a systematic overview of the research landscape in this rapidly growing field is lacking.

**Methods:**

We performed a comprehensive bibliometric analysis of publications on miRNA regulation in pulmonary fibrosis from January 1, 2000, to December 31, 2025, using the Web of Science Core Collection and Scopus databases. After deduplication, a total of 2,093 valid documents (articles and reviews) were analyzed. Multiple analytical tools—VOSviewer (v1.6.20), CiteSpace (v6.4R1), and the bibliometrix R package (v3.2.1)—were employed to examine annual publication trends, collaborative networks (countries, institutions, authors), co-citation clusters, citation bursts, journal dual-map overlays, and keyword evolution.

**Results:**

The annual publication output increased steadily from 2007 to 2021, peaking at 229 papers, then stabilized at 150–193 papers per year (2022–2025). China (889 papers, 38.4%) and the United States (477 papers, 20.6%) were the most productive countries, although United States and European papers exhibited higher average citation impact (United States: 74.79; Germany: 86.49) compared to China (29.28). Nanjing Medical University (43 papers) and the University of Pittsburgh (28 papers) were leading institutions. Author productivity followed Lotka’s Law, with 89.6% of authors publishing only one paper. Co-citation network analysis (1,108 nodes, *Q* = 0.8242, S = 0.9284) revealed well-defined clusters centered on non-coding RNAs, extracellular vesicles, and cellular senescence. Citation burst detection identified 25 key references, with the Raghu G (2022) clinical guideline showing the most sustained influence. Keyword co-occurrence analysis generated six clusters, with the newest frontier (exosomes, extracellular vesicles, mesenchymal stem cells, COVID-19) having average publication years of 2022–2023. Recent keyword bursts included “systematic review” (2021–2023), “immunofluorescence” (2022–2025), and “circular ribonucleic acid” (2023–2025).

**Conclusion:**

This bibliometric analysis provides a systematic mapping of the global research landscape on miRNA regulation in pulmonary fibrosis over the past 26 years. The field has evolved from basic molecular mechanisms toward emerging frontiers including extracellular vesicle-based communication, circular RNAs, and cellular senescence. These findings serve as a valuable resource for researchers seeking to understand current trends, collaborative networks, and future directions in miRNA-targeted pulmonary fibrosis research.

## 1 Introduction

Pulmonary fibrosis represents a heterogeneous group of interstitial lung diseases characterized by progressive scarring of the lung parenchyma, leading to impaired gas exchange, respiratory failure, and ultimately high mortality (1–3). Among them, idiopathic pulmonary fibrosis (IPF) is the most common and devastating form, with a median survival of only 3–5 years after diagnosis (4). Despite the approval of two antifibrotic agents—pirfenidone and nintedanib—these drugs only slow disease progression without offering a cure, underscoring the urgent need for a deeper understanding of the molecular mechanisms driving fibrogenesis and for the development of novel diagnostic biomarkers and therapeutic targets (5, 6). Recent bibliometric analyses have highlighted that targeted therapy research in IPF is increasingly focusing on agents such as TGF-*β* inhibitors and autotaxin inhibitors, reflecting a shift toward more precise molecular interventions (7).

In the past two decades, microRNAs (miRNAs) have emerged as pivotal regulators of gene expression in various biological processes, including inflammation, apoptosis, epithelial-mesenchymal transition (EMT), and extracellular matrix remodeling (8–10). A growing body of evidence has demonstrated that aberrant expression of miRNAs is closely associated with the pathogenesis of pulmonary fibrosis (11). Certain miRNAs, such as miR-21, miR-29, and miR-155, have been shown to modulate key fibrotic pathways involving TGF-*β*, Wnt/*β*-catenin, and PI3K/Akt signaling (12–14). Moreover, the discovery of circulating miRNAs in biofluids has opened new avenues for non-invasive biomarkers, while extracellular vesicle–mediated miRNA delivery has shown promise as a next-generation therapeutic strategy (15).

In recent years, the number of scientific papers on microRNAs (miRNAs) and pulmonary fibrosis has been increasing rapidly. Therefore, it has become extremely difficult to systematically capture the evolution process, knowledge structure, and new frontier areas in this field. Traditional narrative reviews often suffer from subjective bias, whereas bibliometric analysis offers a quantitative and visual approach to map research landscapes, identify core contributors (countries, institutions, authors), reveal collaboration networks, detect citation bursts, and track the transformation of research hotspots over time (16). Notably, bibliometric methods have been increasingly applied to map research trends in pulmonary fibrosis biomarkers, targeted therapies, and related areas, demonstrating the growing recognition of this approach in the fibrosis research community (17).

In this study, we performed a comprehensive bibliometric analysis of the literature on miRNA regulation in pulmonary fibrosis, covering the period from 2000 to 2025, using data retrieved from the Web of Science Core Collection and Scopus. By applying multiple analytical tools (VOSviewer, CiteSpace, and bibliometrix), we aimed to: (1) delineate the annual publication trends; (2) identify the most productive countries, institutions, authors, and journals; (3) visualize co-citation networks and reference bursts; and (4) map keyword co-occurrence and evolution patterns. Our findings provide a systematic overview of the knowledge domain and may serve as a valuable resource for researchers seeking to understand current trends and future directions in miRNA-targeted pulmonary fibrosis research.

## 2 Methods

### 2.1 Materials and methods

The literature data for this study were retrieved from the Web of Science Core Collection (WoSCC) and Scopus databases. A comprehensive search strategy was constructed using subject terms combined with Boolean operators. The search period was set from January 1, 2000, to December 31, 2025, with document types restricted to “Article” and “Review” in English.

The search string for WoSCC was: TS = [(miRNA* OR microRNA* OR “micro RNA*”) AND (“pulmonary fibro*” OR “lung fibro*” OR “idiopathic pulmonary fibro*” OR “interstitial lung disease*” OR “fibrosing alveolitis”)]. Records were exported as “Full Record and Cited References” in plain text format. For Scopus, the search string was: TITLE-ABS-KEY ((miRNA* OR microRNA* OR “micro RNA*”) AND (“pulmonary fibro*” OR “lung fibro*” OR “idiopathic pulmonary fibro*” OR “interstitial lung disease*” OR “fibrosing alveolitis”)), with records exported in CSV format. To avoid the influence of dynamic database updates, all data were exported from both databases on the same date—April 15, 2026—with the search period uniformly set from January 1, 2000, to December 31, 2025.

### 2.2 Inclusion and exclusion criteria

**Figure 1:**
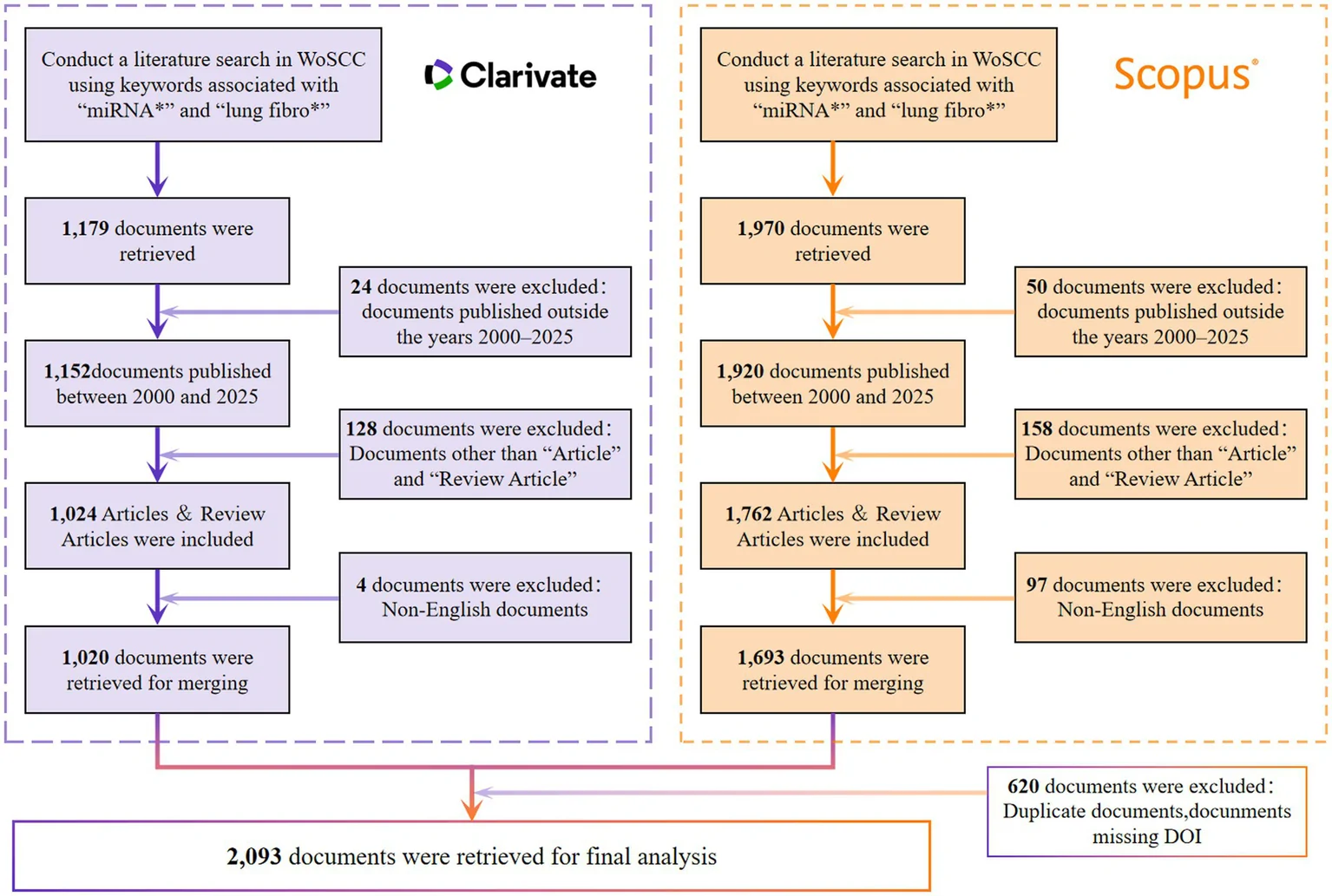
Flowchart of literature inclusion.

The inclusion criteria were as follows: (1) documents indexed as “Article” or “Review” in either WoSCC or Scopus; (2) documents published in English between January 1, 2000, and December 31, 2025; (3) documents whose titles, abstracts, or keywords contained terms related to both miRNAs and pulmonary fibrosis as defined by the search strategy. After deduplication, we manually screened all records to exclude non-relevant document types (e.g., editorials, conference abstracts, letters, corrections). Given that our Boolean search strategy explicitly required the presence of both miRNA-related terms and pulmonary fibrosis-related terms, no additional abstract-level thematic screening was applied. The final dataset comprised 2,093 valid documents. The detailed selection process is illustrated in the flowchart (Figure 1).

### 2.3 Data cleaning and analysis

Python (version 3.11) was utilized to merge and deduplicate the data from WoSCC and Scopus. The script first parsed fields such as institutions and references from the Scopus data and reorganized them to match the WoS plain text format. Duplicate records were identified and removed based on DOI and title matching. A total of 620 duplicates were removed, resulting in a final dataset of 2,093 documents for analysis. During data cleaning, we performed institutional name standardization using a rule-based approach. Common variations (e.g., “Univ” vs. “University,” abbreviations, and parent–child institutional relationships) were consolidated where clearly identifiable.

### 2.4 Bibliometric analysis

Bibliometric analysis and visualization were performed using VOSviewer (v1.6.20), CiteSpace (v6.4R1), Excel (v2024), and the R package “bibliometrix” (v3.2.1). VOSviewer was employed for co-authorship analysis (countries, institutions, and authors) and keyword co-occurrence analysis. CiteSpace was used for citation and keyword burst detection and cluster analysis. The “bibliometrix” package provided supplementary data analysis.

## 3 Results

### 3.1 Bibliometric analysis of annual publications

**Figure 2:**
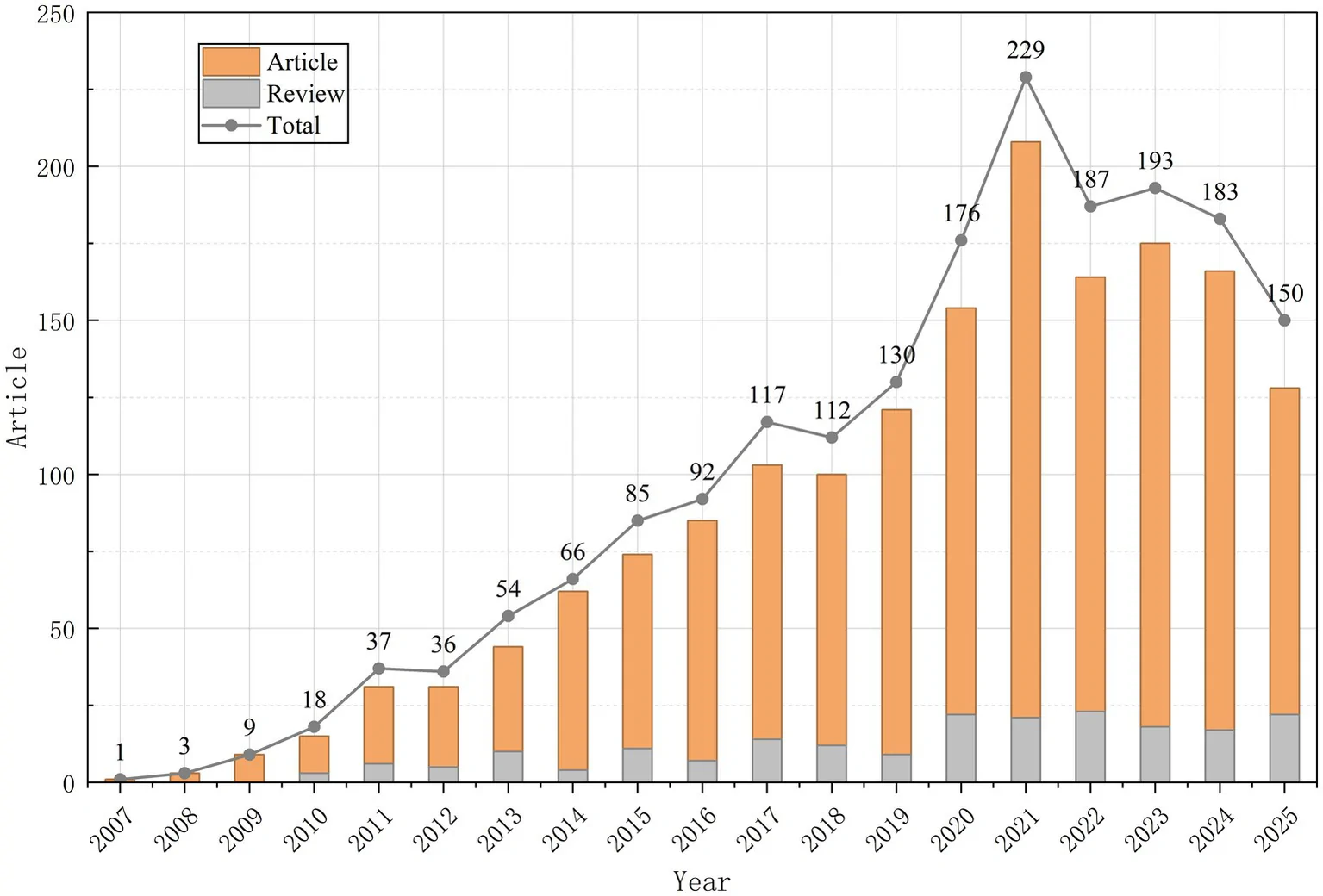
Annual statistical chart of published papers and trend fitting chart. The bar chart shows the number of publications per year (primary y-axis, left). A clear three-phase evolution is observed: Initial accumulation (2007–2012), steady growth (2013–2020), and high-level fluctuation/stabilization (2021–2025). The peak of 229 publications occurred in 2021.

The annual publication volume from 2007 to 2025 (Figure 2) exhibited a trend of significant initial growth followed by high-level fluctuations. The period from 2007 to 2012 represented an initial accumulation stage, with annual publications remaining low (1–37 papers). From 2013 to 2020, the field entered a steady growth phase, with publications increasing from 54 to 176. A peak was reached in 2021 with 229 publications. Between 2022 and 2025, the volume stabilized between 150 and 193 papers. Research articles remained the dominant document type throughout the study period.

### 3.2 Bibliometric analysis of countries and institutions

**Figure 3:**
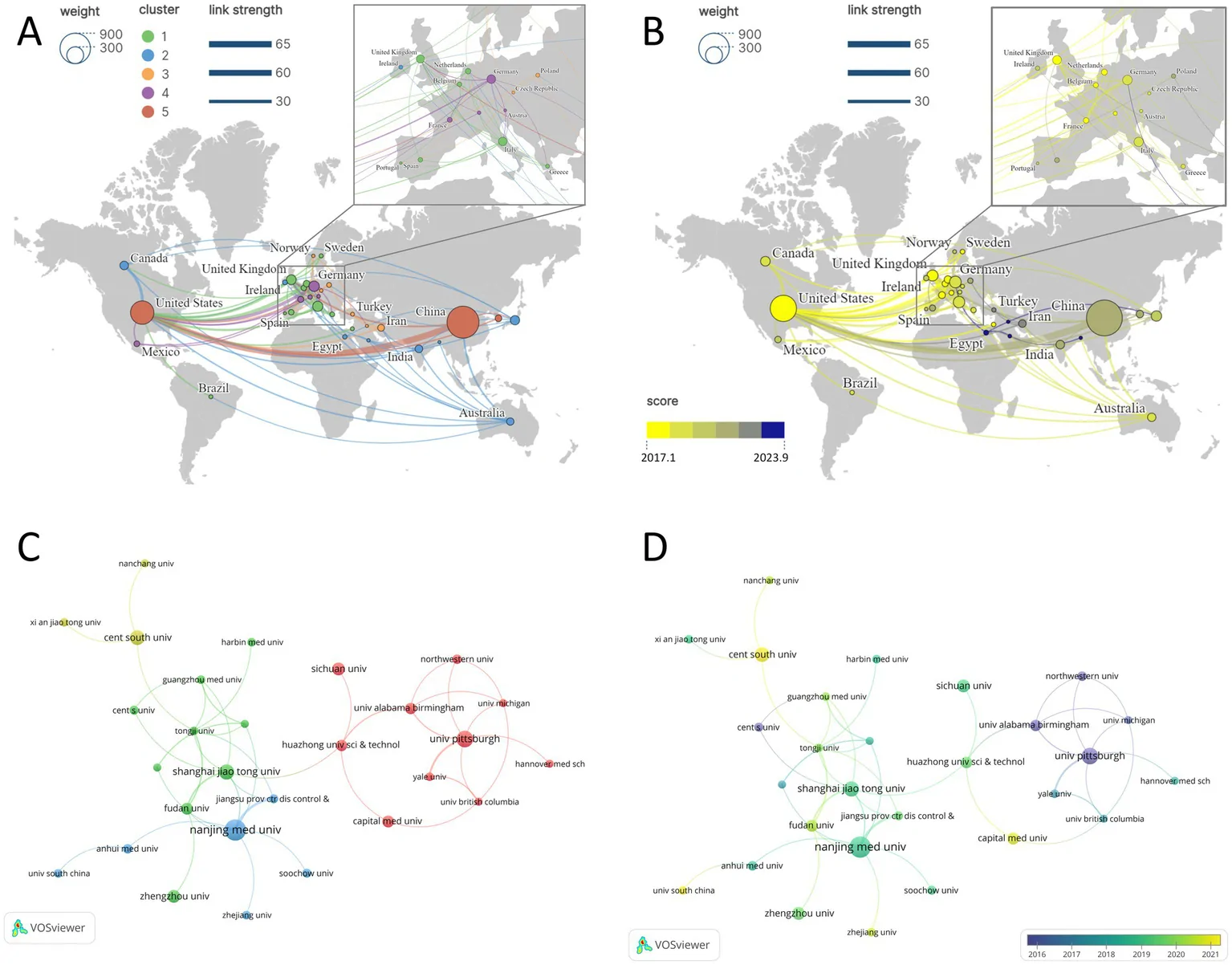
Analysis of countries and institutions publications. **(A)** Network diagram of collaborative relations between the top 32 most productive countries, the node size corresponds to publication volume, the color represents cluster affiliation, and both the color saturation and width of the inter-node connections indicate the strength of collaboration between nodes. **(B)** Co-occurrence overlay map of the top 32 most productive countries, the color brightness of the nodes corresponds to their temporal occurrence, where higher brightness indicates more recently proposed concepts, while lower brightness corresponds to earlier origins. **(C)** Co-occurrence network diagram of the top 28 most productive institutions. **(D)** Co-occurrence overlay map of the top 28 most productive institutions.

Research on Pulmonary fibrosis and miRNA spans 91 countries. The top three contributors—China (889), the United States (477), and Germany (95)—dominate the field, with China and the United States leading significantly (Figure 3A; Tables 1, 2). The country cooperation network comprises five primary clusters. The largest, centered on China, the United States, and South Korea, shows the strongest collaboration between China and the United States (weight 65), alongside high-intensity ties between the United States-United Kingdom (23) and China-United Kingdom (11), reflecting cross-continental radiation. The second cluster, primarily European (United States, Italy, Netherlands, Spain), radiates to Brazil and Israel. A third cluster centered on Germany, France, Switzerland, and Austria exhibits high Central European concentration, with Germany acting as a hub (total link strength 114). Other notable clusters include an Iran-Norway-Poland-Turkey group and a Canada-Australia-India-Japan cross-continental group. Figure 3B indicates that early research was concentrated in Western nations (United States: 2018.09; United Kingdom: 2018.06; Germany: 2018.37), while the later average publication years for China (2020.59) and India (2021.24) highlight their rapid rise over the last 5 years.

**Table 1:** Top 10 most productive countries in terms of publications.

Country	Documents	Citations	Average citations
China	889	26,030	29.28
United States	477	35,675	74.79
Germany	95	8,217	86.49
Italy	90	4,538	50.42
United Kingdom	83	8,734	105.23
Japan	75	2,660	35.47
Canada	64	4,473	69.89
India	51	1,754	34.39
Australia	48	2,714	56.54
Iran	40	1,278	31.95

**Table 2:** Top 10 most productive organizations in terms of publications.

Organization	Documents	Citations	Average citations
Nanjing Med Univ	43	2,115	49.19
Univ Pittsburgh	28	3,089	110.32
Shanghai Jiao Tong Univ	24	1,393	58.04
Cent South Univ	23	526	22.87
Sichuan Univ	19	714	37.58
Zhengzhou Univ	19	676	35.58
Capital Med Univ	17	694	40.82
Fudan Univ	17	515	30.29
Huazhong Univ Sci & Technol	15	806	53.73
Univ Alabama Birmingham	15	3,118	207.87

Co-occurrence analysis included 3,111 institutions. Eight of the top 10 most productive institutions are Chinese, led by Nanjing Medical University (43), Shanghai Jiao Tong University (24), and Central South University (23), highlighting China’s proactive role. The University of Pittsburgh (28) and University of Alabama at Birmingham (UAB, 15) also serve as core nodes. The institutional network forms four clusters: Cluster 1, centered on Pittsburgh, includes Yale (weight 5), UAB, and Michigan, with Huazhong University of Science and Technology and Capital Medical University integrated via cooperation. Cluster 2 features dense Fudan-Shanghai Jiao Tong cooperation (weight 4) and extensive links to Tongji, Nanjing, and Central South universities. Cluster 3 is centered on Nanjing Medical University, showing prominent ties with the Jiangsu CDC (weight 8) and other regional medical universities. Cluster 4 is anchored by Central South University, connecting Nanchang and Xi’an Jiaotong Universities (Figure 3C). The temporal overlay reveals early dominance (2015–2017) by United States institutions (e.g., Pittsburgh: 2015.64; Northwestern: 2015.83). Since 2019, the average publication years for Chinese institutions (e.g., Shanghai Jiao Tong: 2019.13; Central South: 2021.09) have shifted significantly forward, reflecting a rapid catch-up effect and expanding research output (Figure 3D).

### 3.3 Bibliometric analysis of active authors

**Figure 4:**
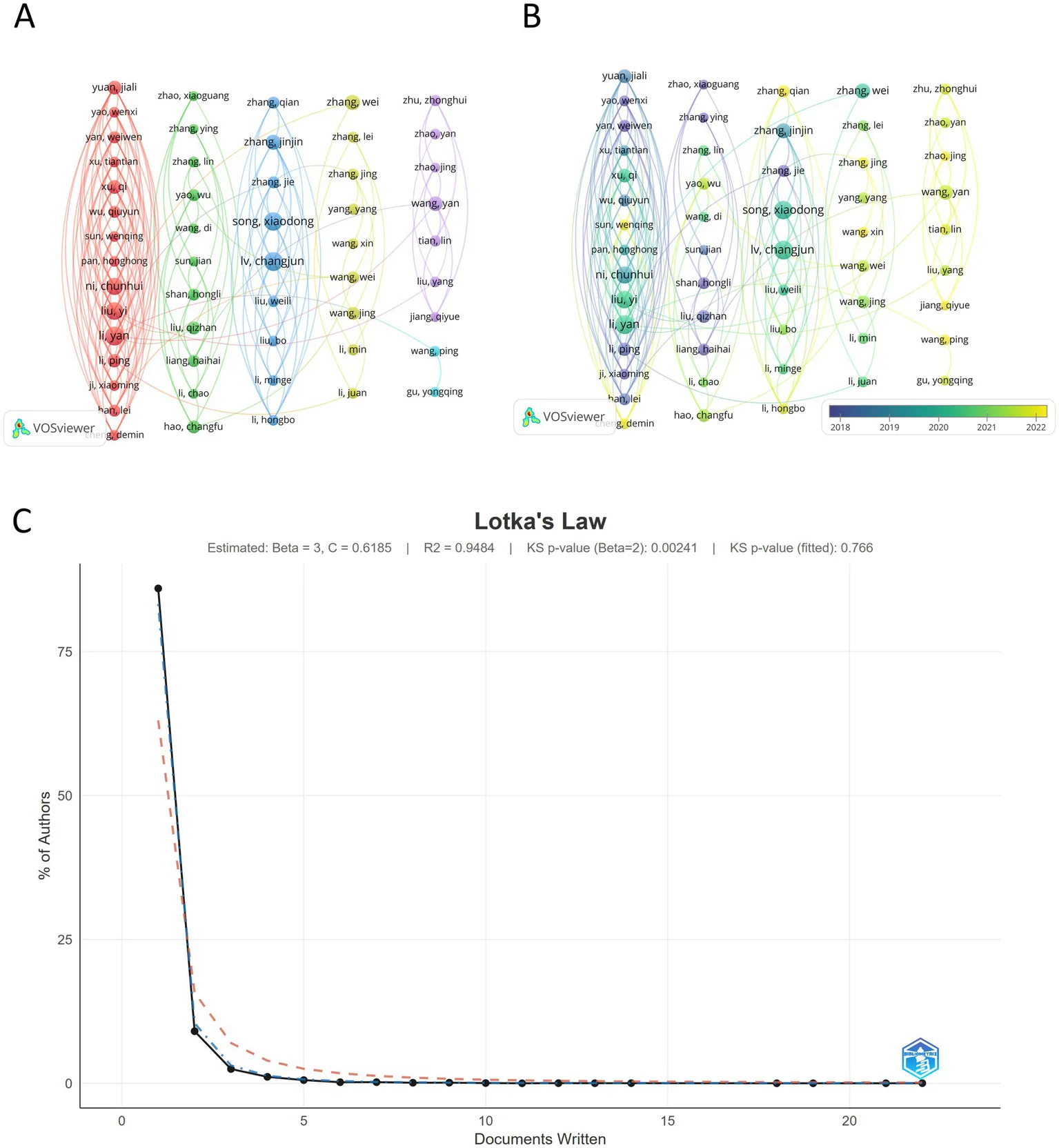
Analysis of authors publications **(A)** Co-authorship map of the top 53 most productive authors, the node size corresponds to publication volume, the color represents cluster affiliation, and both the color saturation and width of the inter-node connections indicate the strength of collaboration between nodes. **(B)** Temporal overlay visualization of the co-authorship network among the most productive authors. Node size represents the number of publications by each author, and the links between nodes indicate co-authorship relationships, with thicker links representing stronger collaboration. Node color represents the average publication year, ranging from purple/blue for authors who were active earlier to green/yellow for authors who became active more recently **(C)** Author productivity through Lotka’s Law, the solid line represents the theoretical distribution, while the dashed line shows the distribution of actual data.

A total of 4,779 core authors are involved in this field, with the distribution of author productivity exhibiting a significant concentration. Lotka’s Law analysis (Figure 4C) reveals that authors with only one publication account for 89.6%, those with two publications represent 7.3%, and those with three or more constitute less than 3%. This suggests that the majority of authors are transient contributors, while a minority of high-productivity authors dominate the knowledge output. The co-authorship network (Figure 4A) identifies six primary collaboration clusters. Based on Table 3, the largest cluster is anchored by Li Y (22 publications, total link strength [TLS]: 100), who forms a dense core circle with Liu Y (19, 90), Ni CH (18, 115), and Xu Q (12, 79), creating a high-output collaborative group. Another high-productivity cluster features a dual-core structure comprised of Lv CJ (22, 70) and Song XD (21, 70), with a collaboration intensity of 21; they maintain high-frequency cooperation with Zhang JJ (14) and Liu WL (8), reflecting a stable research team structure. Cluster 3 is a medium-scale collaboration network centered around Hao CF (10), Liang HH (9), and Shan HL (9). Furthermore, Cluster 5 exhibits tight cooperation among members including Jiang QY, Tian L, Wang Y, and Zhu ZH, where the collaboration intensity between Jiang and both Tian and Zhu reaches 6. The timeline overlay visualization (Figure 4B) shows that early research efforts were concentrated among scholars active between 2016 and 2018, such as Ji XM (2016.86), Yao WX (2017.57), and Yan WW (2017.78), most of whom are core members of Cluster 1. In contrast, authors in Cluster 5, such as Jiang QY (2023.00) and Zhao J (2023.00), have significantly later average publication years, reflecting the recent emergence of new research forces and the gradual formation of novel collaborative subsystems.

**Table 3:** Top 10 most productive authors.

Author	Documents	Citations	Average citations
LI, Yan	22	1,024	46.55
LV, Changjun	22	748	34.00
SONG, Xiaodong	21	748	35.62
LIU, Yi	19	903	47.53
NI, Chunhui	18	984	54.67
ZHANG, Jinjin	14	617	44.07
ZHANG, Wei	14	694	49.57
WANG, Yan	13	328	25.23
XU, Qi	12	476	39.67
YUAN, Jiali	12	689	57.42

### 3.4 References analysis

**Figure 5:**
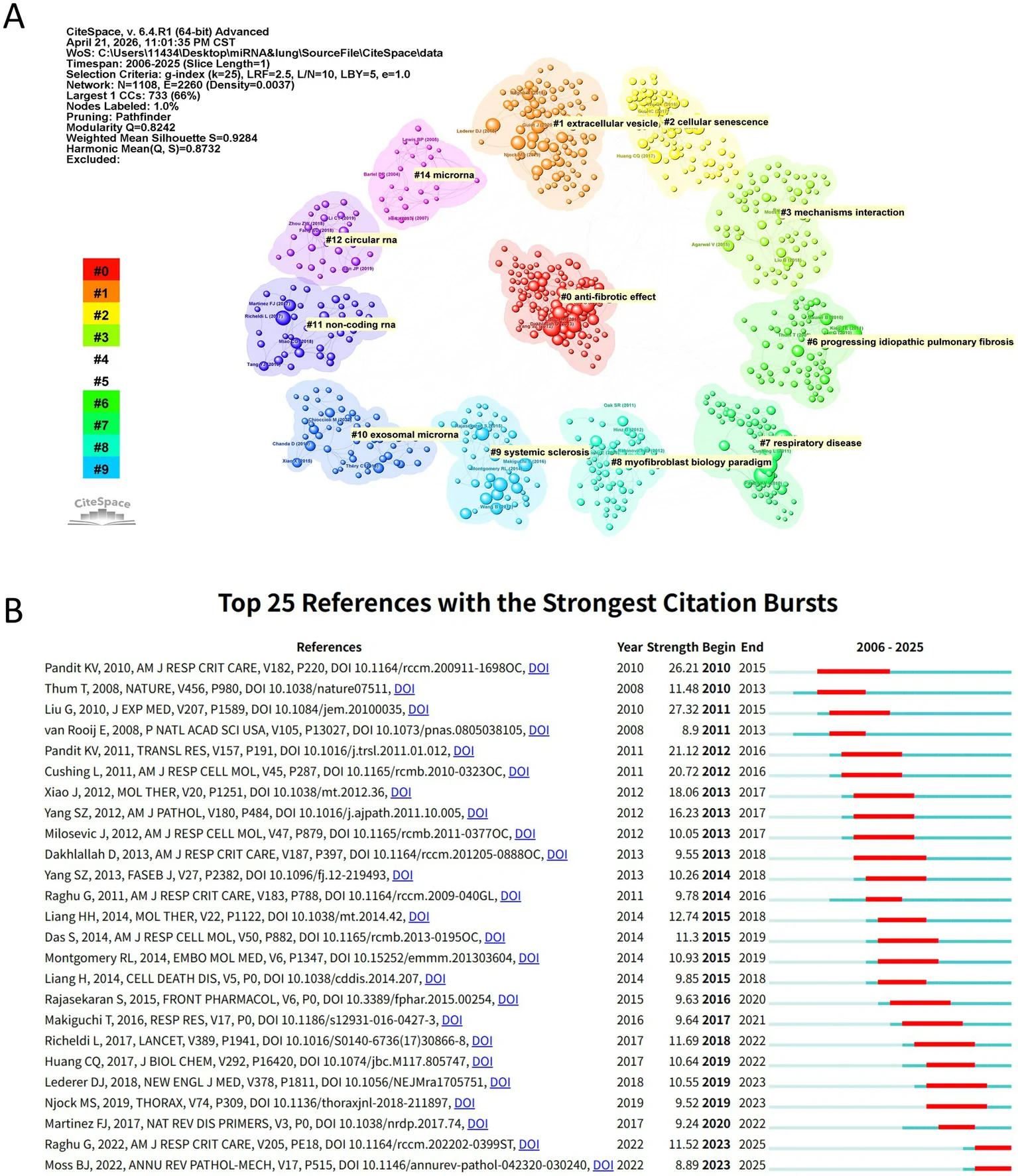
Reference analysis. **(A)** References clustering graph, the figure displays the clustering results of relevant references using the mutual information method, where the black text indicates the theme of each cluster. **(B)** Top 25 references with the strongest citation bursts, the left section displays reference information, while the right area shows the temporal burst of attention each reference received.

A visualization of the document co-citation network from 2006 to 2025 was conducted via CiteSpace, as illustrated in Figure 5A. The network comprises 1,108 nodes and 2,260 links, with a density of 0.0037. The network modularity (Q) is 0.8242, significantly exceeding the 0.3 threshold, and the weighted mean silhouette (S) is 0.9284, indicating a highly significant clustering structure and exceptionally strong intra-cluster homogeneity. Multiple key clusters were identified, reflecting the diversification and deepening of research themes in this field. Cluster #14 (microrna), #11 (non-coding rna), and #12 (circular rna) constitute the core components of the knowledge map, underscoring the dominant role of non-coding RNA regulatory mechanisms in pulmonary fibrosis research. Cluster #0 (anti-fibrotic effect) and #8 (myofibroblast biology paradigm) focus on core pathophysiological processes such as fibroblast activation. Furthermore, the emergence of Cluster #1 (extracellular vesicle) and #2 (cellular senescence) marks the rise of “extracellular vesicle communication” and “cellular senescence” as emerging paradigms for explaining fibrotic progression. Simultaneously, Cluster #6 (progressing idiopathic pulmonary fibrosis) and #9 (systemic sclerosis) reflect a high concentration of research on specific clinical phenotypes and related interstitial lung diseases.

As shown in Figure 5B, citation burst detection identified 25 references with high burst values, which can be categorized into three evolutionary stages. During the early foundational stage (2010–2013), key references such as Thum T (2008, burst strength 11.48), Pandit KV (2010, 26.21), and Liu G (2010, 27.32) emerged, focusing on the regulatory roles of non-coding RNAs in pulmonary fibrosis and establishing the molecular biology foundation for subsequent studies. The mid-term advancement stage (2014–2019) is represented by works such as Yang SZ (2013, 10.26), Liang HH (2014, 12.74), and Das S (2014, 11.30), during which the research focus shifted toward the *in vivo* and *in vitro* functional validation and targeted intervention of specific miRNAs; notably, the review by Lederer DJ (2018, 10.65) exerted an extensive influence on the clinical perception of pulmonary fibrosis. In the recent frontier stage (2019–2025), bursts of references by Njock MS (2019, 9.52), Raghu G (2022, 11.52), and Moss BJ (2022, 8.89) have persisted to the present, marking a new hotspot in knowledge integration and clinical translation discussions. In particular, the clinical guideline by Raghu G (2022) has demonstrated the most enduring influence, indicating that the field is accelerating its transition from mechanistic research to evidence-based practice.

### 3.5 Bibliometric analysis of published journals

**Figure 6:**
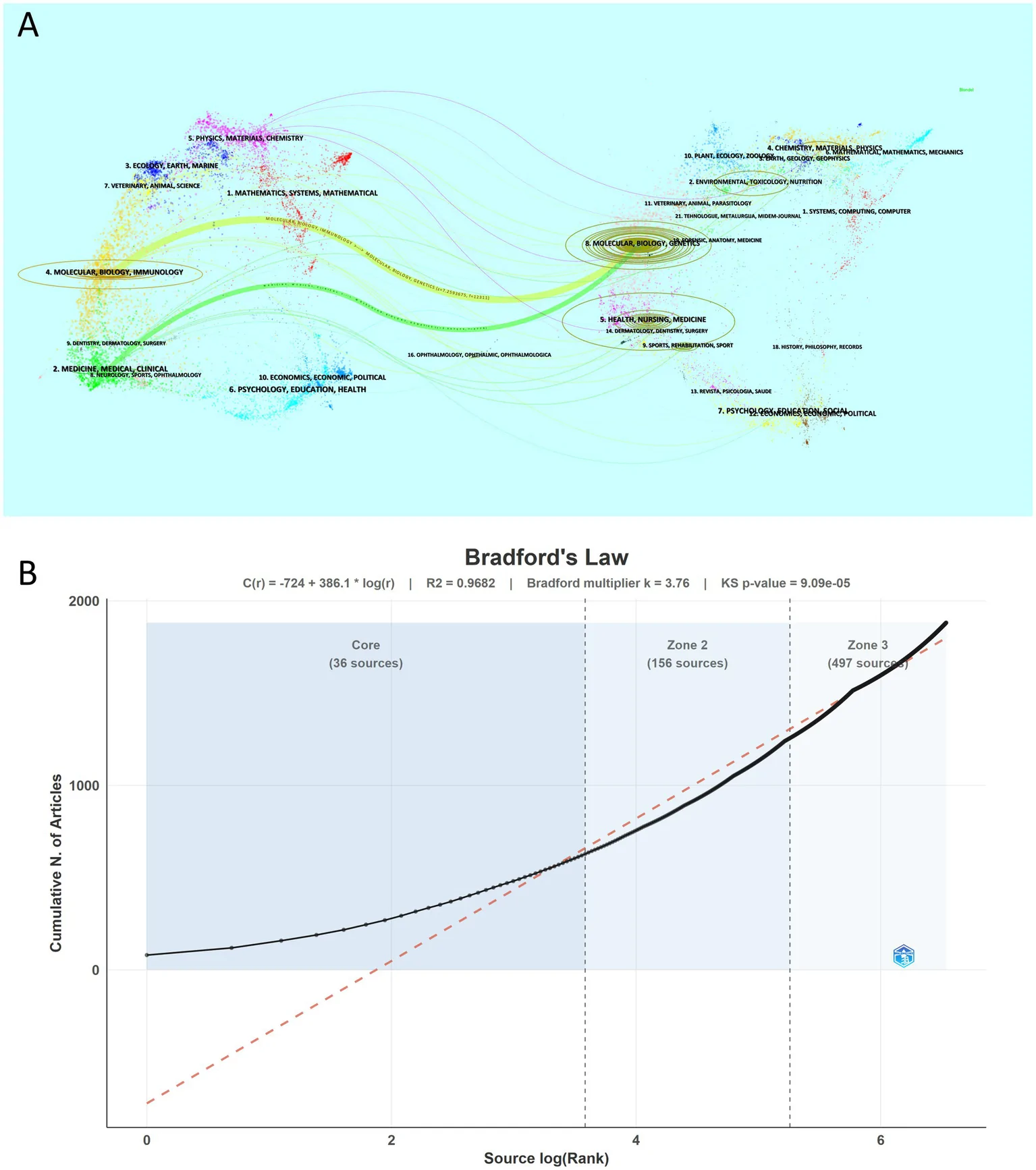
Analysis of sources publications. **(A)** Dual-map overlay of journals: The left side represents the citing journals, the right side shows the cited journals, and the connecting lines in the middle indicate citation relationships between topics. **(B)** Bradford’s law core journal screening and ranking chart: The journals located within the gray area represent the core journals in this field.

The journal dual-map overlay analysis (Figure 6A) elucidates the pathways of knowledge inflow and outflow within this field. The primary citation trajectory exhibits a massive flow from the “Molecular, Biology, Genetics” cluster on the cited side to the “Molecular, Biology, Immunology” cluster on the citing side (z = 7.26, *f* = 12,311). A secondary trajectory originates from the same cited side and flows toward the “Medicine, Medical, Clinical” cluster (z = 2.07, *f* = 3,779). These paths reflect a distinct knowledge migration from basic genetics and molecular mechanism research toward immunological exploration and clinical translation. According to the Bradford’s Law partition model (Figure 6B), although the Kolmogorov–Smirnov (K-S) test indicates a significant deviation between the empirical data and the theoretical Bradford distribution (D = 0.1365, *p* < 0.001), the core-scattering structure remains evident. The core zone comprises only 35 journals (4.2% of the total) yet contributes 702 papers (33.5%). Zone 2 (189 journals, 22.6%) and Zone 3 (611 journals, 73.2%) produced 695 and 696 papers, respectively. This nearly equal distribution of output across the three zones underscores the exceptionally high density of the core zone. Combined with the statistics of highly productive journals (Table 4), the International Journal of Molecular Sciences ranks first with 90 publications (6,321 total citations), followed closely by Frontiers in Immunology with 78 publications (4,331 citations). Other notable contributors include Antioxidants (33 papers) and International Immunopharmacology (31 papers). The 2024 Impact Factors (IF) for these leading journals are primarily concentrated between 4.8 and 6.6, with Antioxidants (6.6) and Frontiers in Immunology (5.9) being particularly prominent. These results demonstrate that research in this field is highly concentrated within high-quality journal clusters specializing in molecular biology, immunology, and pharmacology, forming a stable and high-impact core dissemination platform. It is worth noting that journals with dermatological focuses, such as Archives of Dermatological Research and Journal of Investigative Dermatology, also appear among the contributors. This reflects the substantial body of research on systemic sclerosis-associated interstitial lung disease (SSc-ILD), a pulmonary fibrotic condition commonly published in rheumatology and dermatology journals due to its multi-organ involvement. These publications remain relevant to our topic as they address pulmonary fibrosis mechanisms and miRNA regulation within the broader fibrotic disease spectrum.

**Table 4:** Top 10 most productive journals.

Sources	Documents	Citations	IF^2024^
International Journal of Molecular Sciences	90	6,321	4.90
Frontiers in Immunology	78	4,331	5.90
Antioxidants	33	881	6.60
International Immunopharmacology	31	938	4.81
Frontiers in Pharmacology	30	849	4.80
Cells	29	1,461	5.20
Archives of Dermatological Research	27	504	2.19
Biomolecules	25	1,583	4.80
Journal of Investigative Dermatology	23	782	5.70
Pharmaceutics	21	473	5.50

### 3.6 Keywords analysis

#### 3.6.1 Analysis of keyword co-occurrence

**Figure 7:**
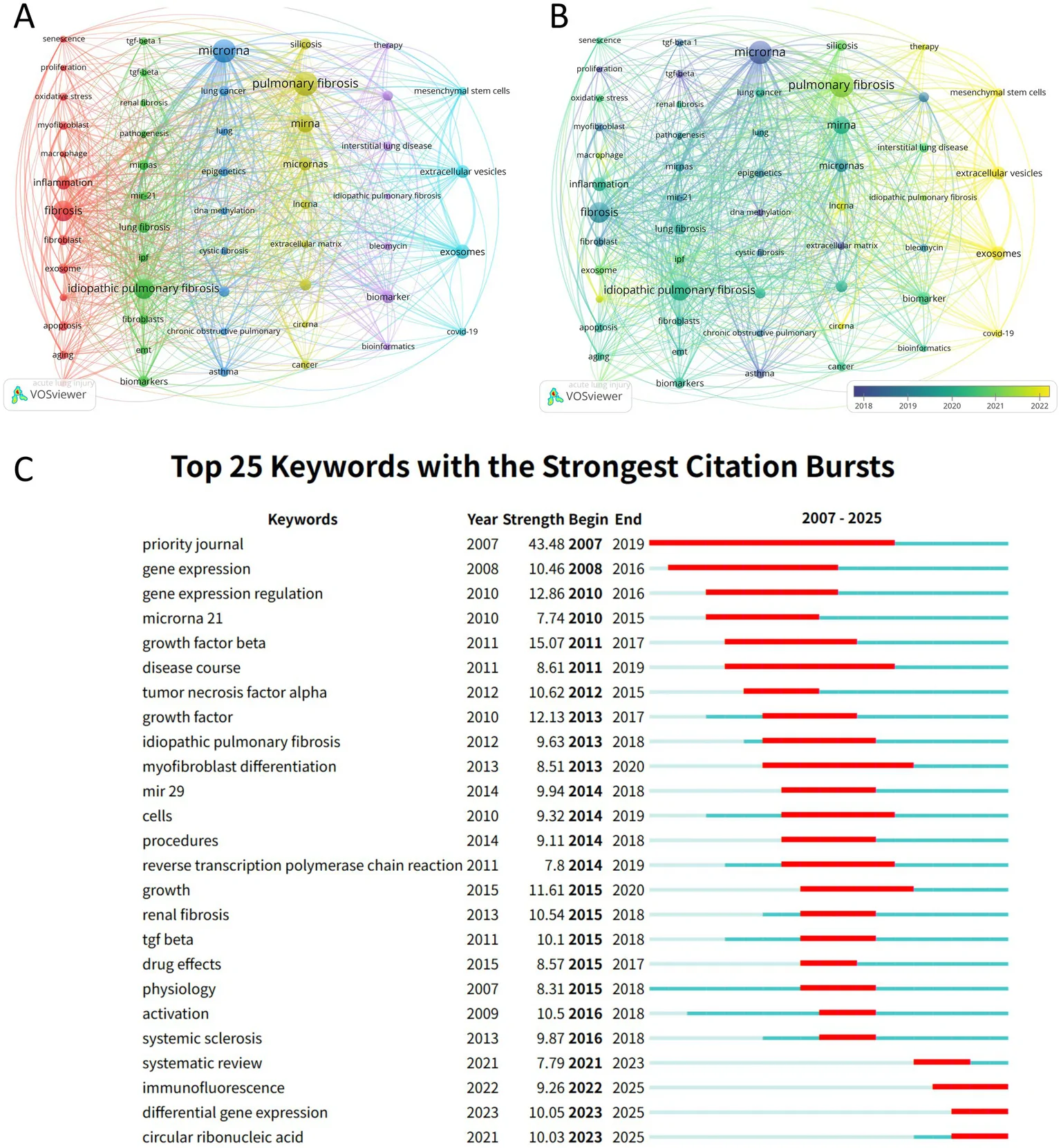
Keyword analysis. **(A)** Co-occurrence network diagram of the top 54 most frequent keywords, the node size corresponds to publication volume, the color represents cluster affiliation, and both the color saturation and width of the inter-node connections indicate the strength of collaboration between nodes **(B)** Co-occurrence overlay map of the top 54 most frequent keywords, the color brightness of the nodes corresponds to their temporal occurrence, where higher brightness indicates more recently proposed concepts, while lower brightness corresponds to earlier origins. **(C)** Top 25 keywords with the strongest citation bursts, the left section displays reference information, while the right area shows the temporal burst of attention each reference received.

A total of 54 high-frequency keywords with a frequency ≥17 were included in this study, and their co-occurrence network is shown in Figure 7A, forming six primary clusters. Cluster 1 is centered on fibrosis (161 times, link strength 233), inflammation (73, 114), and apoptosis (35, 51), closely connecting nodes such as aging, autophagy, and exosome, focusing on the basic pathological mechanisms of pulmonary fibrosis. Cluster 2 aggregates idiopathic pulmonary fibrosis (158, 228), lung fibrosis (52, 70), IPF (32, 48), as well as fibroblasts (38, 70) and EMT (30, 43), embodying research on the clinical and molecular regulation of idiopathic pulmonary fibrosis. Cluster 3 is dominated by microrna (209, 299), interconnecting keywords such as COPD, asthma, lung cancer, and epigenetics, highlighting the pivotal role of non-coding RNAs in various lung diseases. Cluster 4 revolves around pulmonary fibrosis (227, 262), miRNA (115, 177), lncRNA (37, 71), and silicosis (47, 49), reflecting the research landscape of non-coding RNAs involved in fibrotic diseases such as silicosis. Cluster 5 focuses on biomarker (61, 87), systemic sclerosis (38, 71), and interstitial lung disease (36, 51), pointing toward the diagnosis and treatment of systemic sclerosis-associated pulmonary fibrosis. Cluster 6 includes exosomes (81, 134), extracellular vesicles (60, 105), and mesenchymal stem cells (30, 63), and is associated with COVID-19 (25), representing the emerging frontier of extracellular vesicles and mesenchymal stem cell therapy. The temporal overlay map (Figure 7B) shows that the average publication years for nodes such as microrna and epigenetics are earlier (2018–2019), whereas extracellular vesicles (2022.08), mesenchymal stem cells (2022.13), and circRNA (2022.75) in Cluster 6 are significantly later, indicating that the research frontier is shifting from traditional mechanisms toward extracellular vesicles and circular RNAs.

#### 3.6.2 Analysis of keywords citation burst

Keyword burst detection (Figure 7C) further quantifies the evolution of research hotspots. Early burst keywords such as priority journal (2007–2019 strength: 43.48) gene expression (2008–2016) and growth factor beta (2011–2017) were characterized by long durations and laid the foundation for molecular mechanism research. Representative mid-term burst keywords including idiopathic pulmonary fibrosis (2013–2018) myofibroblast differentiation (2013–2020) and renal fibrosis (2015–2018) signify the deepening of fibrosis mechanism research. Recently systematic review (2021–2023) immunofluorescence (2022–2025) and circular ribonucleic acid (2023–2025) have shown sustained bursts with priority journal exhibiting the highest strength (43.48). These results reveal that evidence integration spatial omics technologies and circular RNA regulation constitute the latest frontiers in the field as the research paradigm transitions toward high-resolution molecular phenotypes and evidence-based translation.

## 4 Discussion

In this study, we performed a comprehensive bibliometric analysis of the literature on miRNA regulation in pulmonary fibrosis, covering a 26-year period (2000–2025) with 2,093 valid documents retrieved from the Web of Science Core Collection and Scopus. By integrating multiple analytical tools (VOSviewer, CiteSpace, and the bibliometrix R package), we systematically mapped the research landscape, including publication trends, collaborative networks of countries, institutions and authors, core journals, co-citation clusters, citation bursts, and keyword evolution. Our findings not only highlight the rapid growth and structural characteristics of this field but also reveal the dynamic shift of research frontiers from basic molecular mechanisms toward extracellular vesicle-based communication, circular RNAs, and interest in clinical translation.

The annual publication output showed an overall increasing trend from 2007 to 2025, with a clear inflection point around 2013. This growth trajectory can be divided into three phases: an initial accumulation stage (2007–2012), a steady growth phase (2013–2020), and a high-level fluctuation stage (2021–2025). The peak of 229 papers in 2021 coincides with the global surge in biomedical research resources and the increasing recognition of non-coding RNAs as key regulators in fibrosis. The stable output of 150–193 papers per year between 2022 and 2025 suggests that the field has matured, moving from exploratory discovery to hypothesis-driven and translational research. This pattern is consistent with bibliometric analyses in other fibrotic disease domains, such as liver and renal fibrosis, where a similar “growth-stabilization” trajectory has been observed (18, 19).

China and the United States were the two most productive countries, with 889 and 477 publications, respectively. However, the average number of citations per publication was lower for China (29.28) than for the United States (74.79) and Germany (86.49). The later average publication year of Chinese papers (2020.59 versus 2018.09 for the United States) may have contributed to this difference by providing a shorter citation-accumulation window. Nevertheless, the approximately 2.5-year difference is unlikely to fully explain the magnitude of the citation disparity. Citation impact can also be influenced by publication year, journal distribution, international collaboration, document type, highly cited outliers, and citation practices, none of which were specifically controlled for in the present analysis. Therefore, the observed differences should be interpreted descriptively and should not be considered direct evidence of differences in research quality or originality. The country collaboration network further shows that the China-United States link is the strongest (weight 65), followed by United States-United Kingdom and China-United Kingdom, demonstrating a stable trans-continental collaboration core. European countries (Germany, France, Switzerland, Austria) form a tightly knit cluster, with Germany acting as a regional hub. The temporal overlay indicates that Western countries led early research (United States: 2018.09, United Kingdom: 2018.06), whereas China and India have emerged as major players in the last 5 years. This shift reflects China’s substantial investment in pulmonary fibrosis and miRNA research, as well as the growing scientific output from Asian countries.

At the institutional level, eight of the top ten most productive institutions are Chinese, with Nanjing Medical University ranking first (43 papers). The University of Pittsburgh (28 papers) remains a non-Chinese core node, especially in early years (average publication year 2015.64). The institutional network clusters reveal a pattern of intra-regional cooperation (e.g., Fudan-Shanghai Jiao Tong) and international links (e.g., Huazhong University of Science and Technology with Pittsburgh). The later average publication years of Chinese institutions (e.g., Central South University: 2021.09) confirm their recent rise and expanding influence.

A total of 4,779 core authors were identified, with a highly skewed productivity distribution: 89.6% of authors published only one paper, while authors with three or more papers accounted for less than 3%. This pattern, conforming to Lotka’s Law, is typical of emerging fields where many transient contributors enter but only a few sustained research groups produce the majority of knowledge (20). The co-authorship network showed six clusters, with the largest cluster centered on Li Y, Liu Y, Ni CH, and Xu Q, representing a high-output team. Another dual-core cluster (Lv CJ and Song XD) exhibits intensive collaboration (link strength 21), indicative of a stable and productive research group. The temporal overlay showed that early active authors (e.g., Ji XM, Yao WX) belonged to Cluster 1, while newer authors (Jiang QY, Zhao J) in Cluster 5 have an average publication year around 2023, reflecting the infusion of fresh research forces. These findings highlight the importance of fostering sustained collaboration networks to advance the field, rather than relying on isolated, low-productivity contributors.

The co-citation network (1,108 nodes, 2,260 links) exhibited high modularity (*Q* = 0.8242) and a silhouette value of 0.9284, indicating well-defined and highly homogeneous clusters. The major clusters #14 (microrna), #11 (non-coding rna), and #12 (circular rna) form the core knowledge backbone, confirming that non-coding RNAs—especially miRNAs and, more recently, circular RNAs—are central to the field. Cluster #0 (anti-fibrotic effect) and #8 (myofibroblast biology paradigm) focus on the key pathophysiological processes of fibroblast activation and matrix deposition. Notably, the emergence of Cluster #1 (extracellular vesicle) and #2 (cellular senescence) indicates that “extracellular vesicle communication” and “cellular senescence” have become novel explanatory paradigms for fibrotic progression. This is consistent with recent experimental evidence showing that exosomal miRNAs can mediate cross-talk between epithelial cells, fibroblasts, and immune cells, thereby amplifying fibrotic signals (21).

Citation burst analysis of references identified 25 key papers that have shaped the field. The early foundational stage (2010–2013) included works by Thum T (2008, burst 11.48), Pandit KV (2010, 26.21), and Liu G (2010, 27.32), which established the basic regulatory roles of miRNAs in pulmonary fibrosis. The mid-term advancement stage (2014–2019) was marked by functional validation and targeted intervention studies (e.g., Yang SZ, Liang HH, Das S), as well as the highly influential review by Lederer DJ (2018, burst 10.65) that integrated clinical perspectives. The recent frontier stage (2019–2025) includes bursts from Njock MS (2019), Raghu G (2022, burst 11.52), and Moss BJ (2022, burst 8.89). The persistent burst of the Raghu G (2022) clinical guideline underscores a field-wide transition from mechanistic research toward evidence-based practice and clinical management. This evolution mirrors the broader trend in fibrosis research: from identifying individual miRNAs to understanding complex regulatory networks, and from bench studies to bedside applications.

The journal dual-map overlay revealed two major citation trajectories: a dominant flow from the “Molecular, Biology, Genetics” cited cluster to the “Molecular, Biology, Immunology” citing cluster (*z* = 7.26), and a secondary path toward “Medicine, Medical, Clinical” (*z* = 2.07). This pattern clearly illustrates that basic genetic and molecular mechanism research on miRNAs is being increasingly adopted by immunology-oriented studies and, to a lesser extent, by clinical medicine. The core journal zone (only 35 journals, 4.2% of total) produced 33.5% of all papers, with the International Journal of Molecular Sciences (90 papers) and Frontiers in Immunology (78 papers) as the most prolific outlets. The impact factors of these core journals range between 4.8 and 6.6, indicating that the field is disseminated through high-quality, specialized journals rather than top-tier general medical journals (e.g., NEJM, Lancet). This suggests that miRNA-pulmonary fibrosis research remains largely within the domains of molecular biology, immunology, and pharmacology, with clinical translation still in its early stages.

Keyword co-occurrence analysis generated six clusters each capturing a distinct thematic focus. Cluster 1 (fibrosis inflammation apoptosis) and Cluster 2 (idiopathic pulmonary fibrosis fibroblasts EMT) represent the core pathological mechanisms. Cluster 3 and Cluster 4 emphasize the role of non-coding RNAs (miRNA lncRNA) across various lung diseases including COPD asthma lung cancer and silicosis. Cluster 5 focuses on clinical translation (biomarker systemic sclerosis interstitial lung disease). Importantly Cluster 6 (exosomes extracellular vesicles mesenchymal stem cells COVID-19) represents the newest frontier with average publication years of 2022–2023.

It is important to note that Cluster 3 and Cluster 4, while centered on non-coding RNA regulation, include keywords spanning multiple pulmonary diseases (COPD, asthma, lung cancer, silicosis). This reflects the fact that many miRNA-mediated regulatory pathways are shared across different lung pathologies rather than being strictly fibrosis-specific. Thus, these clusters should be interpreted as representing the broader landscape of miRNA research in pulmonary diseases, from which fibrosis-related studies have emerged as a major sub-domain. In contrast, Cluster 2 (idiopathic pulmonary fibrosis, fibroblasts, EMT) and Cluster 6 (exosomes, extracellular vesicles, mesenchymal stem cells) more directly capture fibrosis-specific mechanisms and therapeutic frontiers. This distinction is important for correctly interpreting the intellectual structure of the field.

Keyword burst detection further quantified this evolution. Early bursts (2007–2019) included “priority journal” “gene expression” and “growth factor beta” reflecting the initial molecular exploration. Mid-term bursts (2013–2020) such as “idiopathic pulmonary fibrosis” “myofibroblast differentiation” and “renal fibrosis” indicate a broadening of the fibrotic disease spectrum. The most recent bursts (2021–2025) include “systematic review” (2021–2023) “immunofluorescence” (2022–2025) and “circular ribonucleic acid” (2023–2025). The high strength of “priority journal” (43.48) reflects the field’s rapid growth and the urgency to publish. Collectively these results demonstrate that the research paradigm is shifting from descriptive gene expression studies toward high-resolution molecular phenotyping (e.g., immunofluorescence spatial omics) evidence synthesis (systematic reviews) and the exploration of novel regulatory RNAs (circRNAs). Extracellular vesicles and mesenchymal stem cell-based therapies are also emerging as translational hotspots partly fueled by the COVID-19 pandemic which highlighted the importance of pulmonary fibrosis as a post-infectious complication.

The emergence of extracellular vesicles and circular RNAs as research frontiers, as evidenced by their recent average publication years (2022–2023) and sustained citation bursts, is grounded in compelling mechanistic evidence. Exosomal miRNAs serve as intercellular communication vectors, transferring regulatory signals between alveolar epithelial cells, fibroblasts, and immune cells within the fibrotic niche. This paracrine signaling mechanism enables the propagation of fibrotic stimuli across cell types, amplifying pathological remodeling beyond the initial injury site. Recent experimental studies have provided direct evidence for this paradigm. Zhou et al. (22) demonstrated that alveolar epithelial cell-derived exosomes activate autophagy through the miR-450b-5p/HDAC7 axis, significantly inhibiting the development of pulmonary fibrosis in both TGF-*β*-induced cellular models and bleomycin-induced mouse models. Similarly, Li et al. (23) found that plasma exosomal miR-15a-5p is markedly downregulated in IPF patients, and its restoration reverses bleomycin-induced autophagy inhibition and fibrosis by directly targeting core fucosylation-modified IGF1R and modulating the PI3K/AKT pathway. Furthermore, mesenchymal stem cell-derived exosomal miR-486-5p has been shown to inhibit TGF-β1-induced pulmonary fibroblast differentiation by targeting FGF9, reducing the expression of fibrotic markers including fibronectin, *α*-SMA, vimentin, and collagens (24). These findings collectively position exosomal miRNAs not merely as passive biomarkers but as active participants and potential therapeutic agents in fibrotic progression. Similarly, circular RNAs, with their greater stability compared to linear RNAs due to their covalently closed loop structure, may function as efficient miRNA sponges, competitively binding to miRNAs and thereby fine-tuning the availability of these regulatory molecules for their target mRNAs. The convergence of these two frontiers—exosomal carriers and circRNA regulators—suggests a complex, multi-layer regulatory architecture that warrants integrated investigation using emerging technologies such as single-cell RNA sequencing and spatial transcriptomics.

Several limitations of this study should be acknowledged. First, although we used two major databases (WoSCC and Scopus), some relevant literature indexed only in other databases (e.g., PubMed Central, Embase, or Chinese databases) might have been omitted. Second, our search strings focused on “miRNA” and “pulmonary fibrosis” related terms, which may have inadvertently excluded studies that use broader non-coding RNA terminology or that investigate fibrosis in other organs but with high relevance to the lung. Third, bibliometric indicators such as citations and impact factors are influenced by many factors (e.g., self-citations, journal policies, field size) and do not directly measure scientific quality. Fourth, the analysis of author collaboration is based on authorship alone and does not capture informal scientific exchanges or multi-disciplinary team structures. Fifth, while we performed basic institutional name standardization during data cleaning, variations in English transliteration of Chinese institutional names may not have been fully consolidated. This could lead to minor underestimation of publication counts and collaboration intensities for certain institutions, and represents a inherent limitation of bibliometric analyses relying on database-exported affiliation strings. Finally, the data export date (April 15, 2026) and the inclusion of 2025 publications may introduce a citation time-lag bias, as newer papers have had less time to accumulate citations. Despite these limitations, our analysis provides a robust and up-to-date overview of the field.

Despite the wealth of mechanistic insights, clinical translation of miRNA-based strategies for pulmonary fibrosis remains in its infancy. Major challenges include the development of stable and tissue-specific miRNA delivery systems, the validation of reliable circulating miRNA panels for early diagnosis and prognosis across multi-center cohorts, and the integration of miRNA biomarkers with existing clinical and imaging parameters. Looking forward, we propose several priority directions: (i) large-scale, multi-center validation of circulating miRNA panels as non-invasive biomarkers; (ii) functional dissection of exosomal miRNA-mediated intercellular communication in the fibrotic niche using single-cell and spatial transcriptomics; (iii) exploration of regulatory networks between miRNAs, circRNAs, and other non-coding RNAs; (iv) development of engineered exosomes or lipid nanoparticles for targeted miRNA-based therapy; and (v) investigation of miRNA regulation in post-COVID-19 pulmonary fibrosis, an emerging clinical challenge that has already begun to shape the research agenda.

## 5 Conclusion

This bibliometric analysis systematically maps the global research landscape of miRNA regulation in pulmonary fibrosis from 2000 to 2025. The field has experienced rapid growth, with China and the United States as the dominant contributors, though United States and European papers exhibit higher average citation impact. Research hotspots have evolved from basic molecular mechanisms (e.g., TGF-*β*, EMT) toward emerging frontiers including extracellular vesicles, cellular senescence, circular RNAs, and growing research interest in clinical translation, as evidenced by recent keyword bursts and co-citation clusters. Our findings provide a valuable reference for understanding the intellectual structure, collaborative networks, and evolving directions in this field, highlighting the need for continued efforts to translate mechanistic insights into non-invasive biomarkers and novel therapeutics.

## Data Availability

The original contributions presented in the study are included in the article/supplementary material, further inquiries can be directed to the corresponding authors.
